# Ellagic Acid Containing Nanostructured Lipid Carriers for Topical Application: A Preliminary Study

**DOI:** 10.3390/molecules25061449

**Published:** 2020-03-23

**Authors:** Supandeep Singh Hallan, Maddalena Sguizzato, Gabriella Pavoni, Anna Baldisserotto, Markus Drechsler, Paolo Mariani, Elisabetta Esposito, Rita Cortesi

**Affiliations:** 1 Department of Chemical and Pharmaceutical Sciences, University of Ferrara, 44121 Ferrara, Italy; hllsnd@unife.it (S.S.H.); sgzmdl@unife.it (M.S.); gabriellapavoni@tiscali.it (G.P.); 2 Department of Life Sciences & Biotechnology, University of Ferrara, 44121 Ferrara, Italy; bldnna@unife.it; 3 Bavarian Polymerinstitute (BPI), University of Bayreuth, 95440 Bayreuth, Germany; Markus.Drechsler@uni-bayreuth.de; 4 Department of Life and Environmental Sciences, Polytechnic University of Marche, 60131 Ancona, Italy; p.mariani@staff.univpm.it

**Keywords:** nanostructured lipid carriers (NLCs), lipid-based nanosystems, phytopharmaceutics, ellagic acid, antioxidant activity

## Abstract

Ellagic acid (EA) is a potent antioxidant substance of natural origin characterized by poor biopharmaceutical properties and low solubility in water that limit its use. The aim of the present study was to develop lipid-based nanoparticle formulations able to encapsulate EA for dermal delivery. The EA-loaded nanoparticles were prepared using two different lipid compositions, namely tristearin/tricaprylin (NLC-EA1) and tristearin/labrasol (NLC-EA2). The influence of formulations on size, entrapment efficiency, and stability of EA-loaded nanoparticles was investigated. Cryo-TEM and small-angle X-ray scattering (SAXS) analyses showed that no morphological differences are evident among all the types of loaded and unloaded nanostructured lipid carriers (NLCs). The macroscopic aspect of both NLC-EA1 and NLC-EA2 did not change with time. No difference in size was appreciable between empty and drug-containing NLC, thus the nanoparticle diameter was not affected by the presence of EA and in general no variations of the diameters occurred during this time. The entrapment efficiency of both EA-loaded nanoparticles was almost quantitative. In addition, NLC-EA1 maintained EA stability for almost two months, while NLC-EA2 up to 40 days. FRAP (Ferric reducing ability of plasma) assay showed an antioxidant activity around 60% for both the loaded NLC, as compared to the solution. Although both types of NLC are characterized by some toxicity on HaCaT cells, NLC-EA1 are less cytotoxic than NLC-EA2. Taken together these results demonstrated that the inclusion of EA within NLC could improve the water solubility, allowing for a reduction of the dosage. Moreover, both types of NLC-EA maintained a high antioxidant effect and low toxicity.

## 1. Introduction

Phytopharmaceuticals are pharmaceuticals derived from botanicals. Ellagic acid (EA) is a phytopharmaceutical substance found in many fruits and plants such as raspberries, strawberries, pomegranates, blackberries, and many other plant foods. EA (2,3,7,8-tetrahydroxy-chromeno [5,4,3-cde]chromene-5,10-dione) (Table 1) is the dimeric derivative of gallic acid and has a significant attractiveness in food supplements because of its potentially beneficial effects against a wide range of diseases [1]. Various studies indicate that EA possesses antimutagenic, antiagenic, antioxidant, and anti-inflammatory activity in bacterial and mammalian systems [2,3,4]. In addition, EA has proven to be an efficient skin whitener and suppressor of pigmentation. In addition to its anti-oxidant activities, EA also is cytotoxic towards different types of cancer cells, such as osteogenic sarcoma, tongue, pancreatic, leukemic, neuroblastoma, breast, prostate gland, and colon cells [5,6,7,8,9,10,11], and possesses anti-inflammatory, anti-bacterial, anti-angiogenesis, anti-atherosclerosis, anti-hyperglycemic, antihypertensive, and cardioprotective effects [1,12,13,14]

However, EA has found limited use in therapeutic applications due to its low water solubility (around 9.7 μg/mL) and permeability (class IV of the Biopharmaceutics Classification System) [15,16]. When orally administered EA is poorly absorbed due to low aqueous solubility, metabolism in the gastrointestinal tract [17], first pass effect and irreversible binding to cellular DNA and proteins problems.

Incorporation of drugs in lipid nanoparticles is a smart approach to overcome bioavailability [18]. Among lipid-based colloids, solid lipid nanoparticles (SLNs) and nanostructured lipid carriers (NLCs) can be mentioned. SLNs are generally produced using solid lipids stabilized by the presence of surfactants dispersed in the aqueous phase. As a drawback, during lipid crystallization of the solid lipid core of these particles may lead to leakage of the included active compound, thus influencing the encapsulation efficiency. NLCs were developed to overcome these drawbacks by mean of a partial replacement of the solid lipid with a fluid lipid. In this way the lipid core matrix becomes less ordered as compared to SLNs and can accommodate high amounts of active compound reducing loss problems.

The main advantages of both SLNs and NLCs are their ability to incorporate active compounds, improve stability and bioavailability of the entrapped molecules, possibly control release and targeting together with safety, low cost of production, and easy scaling-up. Due to their great versatility, biodegradability, biocompatibility, and targeting, the lipid-based nanoparticles have been used for several administration routes, such as oral, parenteral, ocular [19], and topical [20,21]. In particular, topical administration of active compounds loaded onto SLNs or NLCs could prevent their systemic absorption and hence, side effects. Furthermore, their small size ensures a close contact with the stratum corneum, facilitating drug skin penetration.

On the other hand, common disadvantages of SLN include particle aggregation, particle growth, unexpected dynamics of polymorphic transition, burst drug release, and low incorporation capacities. Concerning this last drawback, it can be underlined that triglycerides are known to crystallize mainly in three polymorphic forms which transform monotrophically from α, via β′, to β [22]. During storage, SLN triglycerides are subjected to a shift into low energy and more ordered β modification, which cause a reduction of imperfections in the crystal lattice and consequently, drug expulsion. When SLNs are formulated with mono acid glyceride, such as tristearin, the drug loading is limited, and drug expulsion occurs within short times. 

Instead, NLCs are composed of a solid lipid matrix with a certain content of a liquid lipid phase able to better solubilize drugs as compared to solid lipids. The liquid lipids form droplets within the solid lipid particles matrix, providing a high incorporation capacity and a control of drug release. Therefore, NLCs, which are solid but not crystalline, overcome the drawback of drug expulsion. Indeed, the use of mixtures of solid and liquid lipids allows for the obtaining of nanoparticles that become solid after cooling but do not crystallize [23]. In these structures, the oily nano-compartments are surrounded by a solid matrix and the solid matrix of the lipid nanoparticle contains tiny liquid nano-compartments of oil; this peculiar lipid organization increases drug solubility, leading to an enhancement of the drug loading capacity. In addition, analyzing the production methods of SLNs, it was observed that too high of a concentration of the drug in the molten lipidic phase could lead to an immediate drug expulsion during the cooling process or to a dilution in the cold water.

For our studies, we analyzed two different blends of solid and liquid lipids. The first one was based on the use of a mixture of the liquid caprylic/capric triglycerides (Miglyol) and the solid tristearin. In the second case, a mixture of caprylocaproyl macrogol-8 glyceride (Labrasol) and tristearin was used to produce NLCs. In the hot state the two lipids form one phase and during the cooling process a phase separation occurs, leading to inclusion of small oily droplets in the solid matrix. Taking into account these assumptions, the present paper will describe the preparation, characterization, and preliminary in vitro studies of EA-containing NLCs for dermatologic purposes. 

## 2. Results and Discussion

### 2.1. Production and Characterization of NLC-EA Dispersions

The NLC-EA composed as reported in Table 2, were obtained by dispersing the lipid phase in the aqueous phase under sonication [23] achieving stable and homogenous dispersions. The corresponding empty NLC, namely e-NLC1 and e-NLC2, were obtained using the same procedure apart for the addition of EA. As already known, a fluid lipid (i.e., tricaprylin or labrasol) when mixed to solid lipids allows for the formation of solid particles homogenously embedded with fluid compartments. However, during the preparation almost 4% by weight of total used lipid phase was lost on the vessel and less than 1% gave rise to the formation of agglomerates. 

After production, nanoparticles were characterized in terms of dimensions and morphology. Dimensions and size distribution of the produced NLCs were determined by mean of photon correlation spectroscopy (PCS). The analyses were made immediately after preparation and periodically at regular intervals in order to investigate the stability of nanoparticles by time. Table 3 summarizes the obtained values of mean diameters and polydispersity.

Analyzing the obtained values, it should be noted that in general no variations of the diameters occurred during this time. Some differences in size are appreciable between empty and drug-containing nanoparticles only in the case of NLC1, which shows an increase from 116.5 nm to 195.7 nm in the presence of EA. However, concerning the polydispersity indexes, empty NLCs showed a reduction during time while EA-loaded NLCs showed no great variations, indicating that NLCs maintain up to two months their monomodal dimensional distribution.

**Figure 1:**
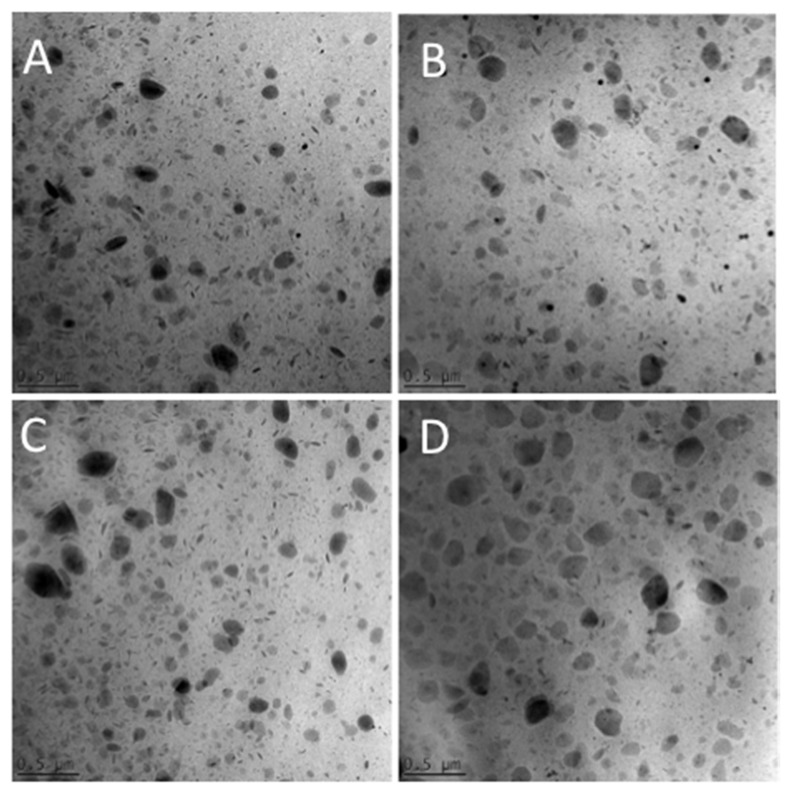
Cryo-transmission electron microscopy (cryo-TEM) images of e-NLC1 (**A**), NLC-EA1 (**B**), e-NLC2 (**C**), and NLC-EA2 (**D**).

Cryo-transmission electron microscopy allows for the study of the morphology of the produced NLCs. Figure 1 depicts the cryo-TEM images of e-NLC1, e-NLC2, NLC-EA1, and NLC-EA2. It is evident that no morphological differences are evident among the considered types of NLCs. Notably, some large and flat particles are detectable together with deformed, elongated, and circular platelet-like or elliptical shaped nanoparticles depending on their position with respect to the site of observation, namely from the top to edge-on view or intermediate positions [24,25].

**Figure 2:**
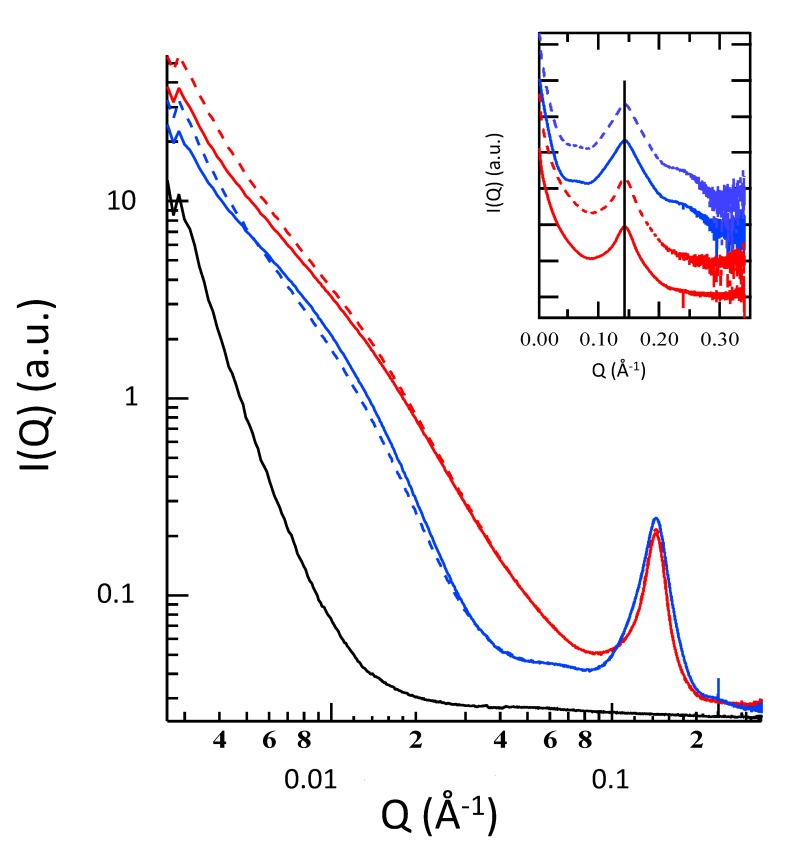
Small-angle X-ray scattering (SAXS) profiles of e-NLC1 (full blue line), e-NLC2 (full red line), NLC-EA1 (dotted blue line), NLC-EA2 (dotted red line). The signal of sole water is indicated with a black line. The inset shows the SAXS profiles after the subtraction of the water contribution. Here, the curves are scaled for clarity. The vertical black line indicates the constant position of the Bragg peaks.

The inner structural features of NLC, NLC-EA1, and NLC-EA2 were investigated by small-angle X-ray scattering (SAXS). Results are reported in Figure 2 and clearly indicate that NLC-EA1 and NLC-EA2 nanoparticles are characterized by a lamellar organization of the inner matrix, both in the presence or absence of EA. Particularly, SAXS profiles show a Bragg peak at Q = 0.143 Å^−1^, which corresponds to a lamellar structure with a repeat distance (which measures the sum of the bilayer thickness and the thickness of the water layer separating two adjacent bilayers) of 43.9 Å. Indeed, the four preparations are very similar, therefore it can be asserted that neither the different lipid composition nor the presence of EA modify the structural organization of lipid nanoparticles. Note that considering the very low solubility of EA in water, data suggests a solubilization of the drug inside the paraffinic region of the lipid layer.

### 2.2. Efficiency of Drug Encapsulation and Shelf-Life

The amount of drug encapsulated in NLCs with respect to the total amount used for the preparation, was evaluated by high-performance liquid chromatography (HPLC) using a reversed-phase column as described in the experimental section. The amount of entrapped EA was determined after centrifugation by dissolving in a known amount of ethanol the lipid phase, while the amount of free EA was determined in the filtrated aqueous phase. The content of EA in both fractions was calculated by comparison with a standard solution. Particularly, EA content in the produced NLCs was calculated as a function of time and expressed as a percentage of the total amount used for the preparation.

As reported in Table 4, drug recovery after NLC production was almost quantitative as compared to the total amount used for the preparation. In the aqueous fraction the amount of EA was always below the detection limit of the analytical method employed, indicating a high encapsulation efficiency of loaded NLC. Indeed, the drug encapsulation was 91.50 ± 2.42% in the case of NLC-EA1 and 96.61 ± 3.67 % in the case of NLC-EA2 dispersions.

Shelf life stability was calculated plotting Log (EA residual content, % with respect to drug content at time 0) against time, obtaining first order kinetics (data not shown). From the slopes (m) obtained by linear regression, the time at which the drug concentration lost 10% and 50%, namely shelf life (*t*_90_) and half-life (*t*_1/2_) respectively, was calculated and reported in Table 4. All data were statistically significant (*p* < 0.0001). 

It was found that EA in solution decomposes quickly (*t*_1/2_ being 56 days), while NLCs are able to increase the protection of EA as compared to the solution with different efficiency. Indeed NLC-EA1 maintain 90% of EA stability for almost two months (57 days), whilst for NLC-EA2, *t*_90_ is around 40 days. The *t*_1/2_ values reach more than one year for NLC-EA1 (378 days) and 8.5 months for NLC-EA2, increasing the stability of EA 6.72- and 4.59-fold as compared to the solution. 

The macroscopic aspect of both NLC-EA1 and NLC-EA2 did not change by time. Notably, no phase separation phenomena, settling of particles, and aggregate formation were evident after three months from production.

### 2.3. In Vitro Experiments

#### 2.3.1. Antioxidant Activity

It is well known that various pathophysiological processes are due to the presence of free radicals, thus the antioxidant intervention is of pivotal importance. Indeed, the beneficial effects of polyphenols on human skin are largely described, such as antioxidant, anti-aging, anti-inflammatory and anti-cancer activities [26,27,28]. In this view, both EA-containing NLC formulations were subjected to two different tests to evaluate their antioxidant capacity as compared to the active solution and the empty NLC. The chosen tests were DPPH (2,2-Diphenyl-1-picrylhydrazyl) and FRAP (Ferric reducing ability of plasma).

It has to be underlined that in both cases it was impossible to test the activity of empty NLCs because the addition of their dimethyl sulfoxide (DMSO) solution to the radical or FRAP mixture gave rise to the formation of a certain opalescence or precipitation, respectively. As for the NLCs containing EA, the data summarized in Table 5 relate to extremely diluted solutions that did not give great opalescence or precipitation problems in the test media. However, unlike EA that is perfectly soluble in the chosen solvent (DMSO), the two NLCs in the same solvent appeared as suspensions.

In light of these observations, the antioxidant activity data reported in Table 5 indicate for both types of NLC-EA a lower activity with respect to EA solution. In particular, regarding the FRAP assay, the antioxidant activity of both loaded NLCs is around 60% as compared to the solution. This behavior could be possibly ascribed to a combination of events. Firstly the recovered amount of EA within the formulation is lower as compared to the solution being comprised between 91%–96% (Table 4) and the amount of solubilized EA in the reaction environment. More precisely, the EA dispersed within solid NLCs can be in solid form, therefore it is not readily available in the useful form to express the antioxidant activity. In other words, there is a lag time in which EA must pass from the solid to the solubilized form in solution capable of interacting with ferric ions. Thus, these conditions may affect the actual EA concentrations useful for the determination of antioxidant activity, but in the meantime they give good results about the potential activities of these formulations. Indeed, the rate of EA dissolution could possibly influence a lasting antioxidant activity during time. On the other hand, the values obtained with the DPPH assay are quite far from those expected, certainly due to the incompatibility of the DMSO solvent with the test methodology [29,30]. The profile emerged from these tests underlines how the two new formulations preserve the excellent antioxidant capacity of the active.

#### 2.3.2. EA Diffusion from NLC

To evaluate the release of EA from NLC-EA1 and NLC-EA2 formulations, Franz-cells associated to nylon membrane were used. Particularly, two different pH values were considered for the receiving phase constituted of phosphate buffer, namely pH 7.4 and 5.5. Furthermore, to establish the sink conditions and promote EA solubilization, 30% ethanol by volume was added to the receiving phase [31,32]. Moreover, it has to be underlined that due to the poor water-solubility of EA, the comparative EA solution used for diffusion release experiments was made in DMSO. 

**Figure 3:**
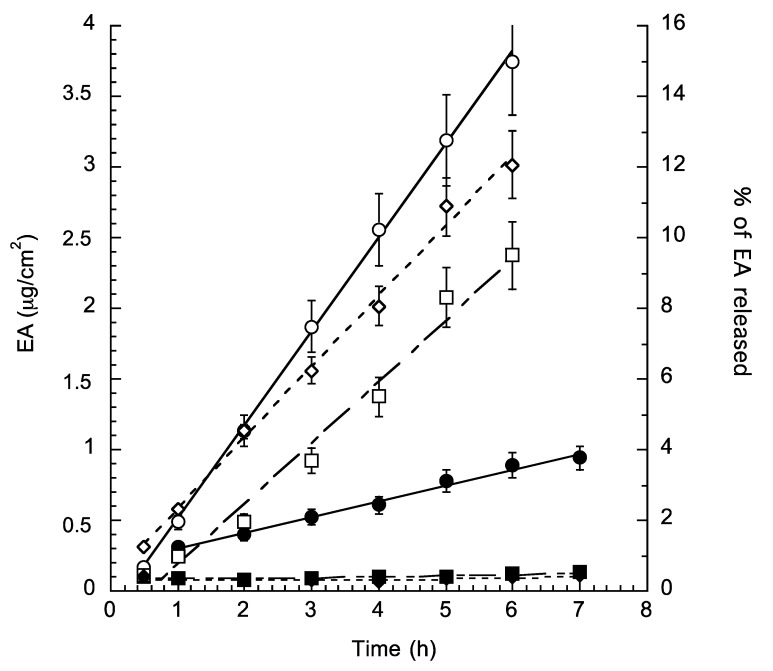
In vitro diffusion kinetics of EA from DMSO solution (circles) or NLC-EA1 (squares) and NLC-EA2 (diamonds) as determined by Franz cells associated to nylon membranes. Experiments were conducted in phosphate buffer at different pH, namely 7.4 (closed symbols) and 5.5 (open symbols). Data are the mean of four independent experiments ± s.d.

Figure 3 reports the diffusion release profiles of EA from solution and both types of NLCs. The amount of EA that penetrated through the membrane per unit area was plotted against time and the slopes, which represent the steady state fluxes, and were calculated by linear regression. The calculated regression coefficients squared were higher than 0.96. The slopes were then substituted into Equation (6) for the determination of normalized fluxes (Jn) and the results of these calculations are reported in Table 6. 

Particularly, in agreement with the scarce water solubility of EA, the Jn values were in general very low at 0.003 and 0.663. Notwithstanding these results, from the obtained profiles it is evident that the two NLC systems displayed a similar behavior in controlling EA release. Moreover, as expected, the influence of the pH on the receiving phase is appreciable. Indeed, as indicated by literature, pH influences the release, solubility, and permeation of acidic drugs [33,34,35,36]. It is interesting to see that at pH typical of skin surface, which ideally should be slightly acidic being comprised in the acidic range from pH 4.0 to 7.0 [33], the release of EA is higher as compared to the same formulation tested at neutral pH (i.e., pH 7.4) (see Figure 3 and Table 5). This result could be noteworthy for a potential topical application of NLC-EA onto the skin. 

#### 2.3.3. Cytotoxicity Studies

**Figure 4:**
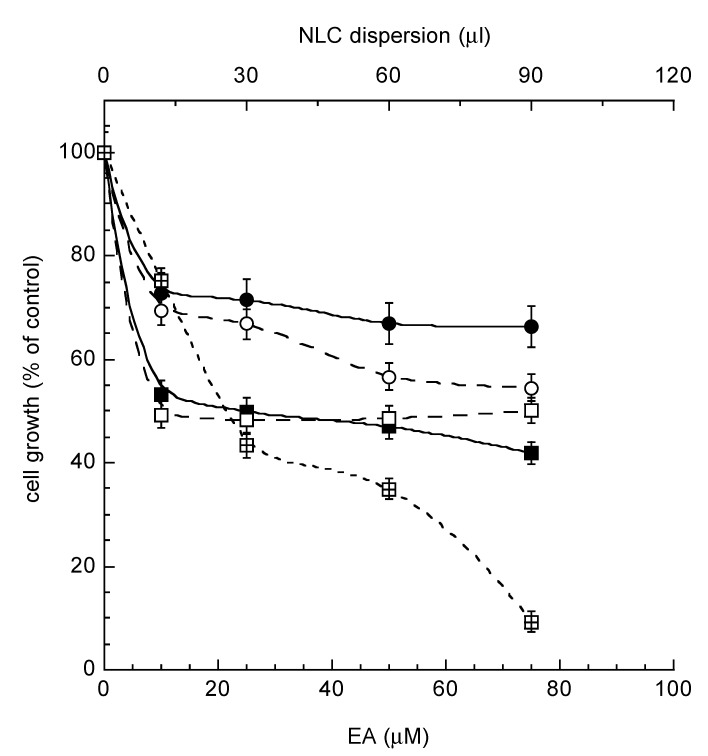
In vitro antiproliferative effect on HaCaT cells of EA in DMSO solution (crossed squares), e-NLC1 (closed circles), e-NLC2 (closed squares), NLC-EA1 (open circles), and NLC-EA2 (open squares). Data are the mean of three independent experiments ± s.d. conducted in triplicate. *p-*values are always <0.01.

It is well known that lipid nanocarriers can improve solubilization and stabilization of drug molecules, thus influencing the pharmacokinetics of drugs in reason of the different distribution after systemic administration [37]. Moreover, lipids are physiological safe compounds as components of many natural food sources and therefore present metabolic pathways for their degradation. In addition, due to the promising results concerning EA diffusion and antioxidant activity, the in vitro activity of the produced formulation was further investigated. Particularly, cytotoxicity was assessed by the colorimetric MTT assay (3-(4,5-dimethylthiazol-2-yl)-2,5- diphenyltetrazolium bromide) on HaCaT cells comparing the activity of EA-loaded NLCs to that of EA in DMSO solution. The obtained results are graphically shown in Figure 4. 

It should be underlined that, although both types of NLCs are characterized by some toxicity, NLC1 are less cytotoxic than NLC2. Furthermore, the presence of EA does not heavily influence the cytotoxicity of these formulations. On the other hand, a result that confirms our initial hypothesis is that both these formulations have a reduced cytotoxicity as compared to that of the DMSO solution, allowing us to propose them as a possible vehicle for EA.

## 3. Materials and Methods

### 3.1. Materials

Ellagic acid was purchased from Sigma-Aldrich. Miglyol 812N (tricaprylin; C8/C10 fatty acid triglycerides; caprylic/capric triglycerides; 1,2,3-propanetriyl ester caprylic acid; caprylic acid, 1,2,3-propanetriyl ester; glycerol trioctanoate; glyceryl tricaprylate; octanoic acid, 1,2,3propanetriyl ester) was a gift of Cremer Oleo Division (Witten, Germany). Labrasol^®^ (caprylocaproyl macrogol-8 glyceride; PEG-8 caprylic/capric glycerides) was purchased form Gattefossé (Saint-Priest, France). Tristearin (Propane-1,2,3-triyltrioctadecanoate; 1,3-Di(octadecanoyloxy)propan-2-yloctadecanoate; stearic triglyceride; glyceryl stearate), poloxamer 188 (methyl-oxirane polymer, 75:30), and all other solvents and materials were provided by Merck (Milano, Italy). 

### 3.2. NLC Preparation

NLCs were prepared by hot homogenization and ultrasonication. Shortly, a lipid mixture composed as reported in Table 2 constituting the 5% by weight as compared to the total weight of dispersions, was melted at 70 °C. Then poloxamer 188 aqueous solution (2.5% *w*/*w*) was added at the same temperature under 15,000 rpm homogenization for 1 min (Ultra Turrax T25, IKA-Werke GmbH & Co. KG, Staufen, Germany). Afterwards the obtained emulsion was ultrasonicated (MicrosonTM, Ultrasonic cell Disruptor) at 7 kHz for 15 min and cooled down to room temperature. 

EA-containing NLCs (NLC-EA) were prepared by adding a DMSO solution of the drug to the molten lipid mixture at 70 °C. Afterwards the production protocol proceeded with the addition of the poloxamer aqueous phase as above described. NLC dispersions were stored at room temperature.

### 3.3. NLC Characterization 

#### 3.3.1. Cryo-Transmission Electron Microscopy (Cryo-TEM)

Samples of 2 µl droplets were vitrified on a lacey carbon filmed copper grid (Science Services, Munich), by insufflation of the mean of air plasma glow discharge (Solarus 950, Gatan Inc., Munich, Germany) for 30 s. After removing the liquid by means of blotting paper, the specimen was frozen by immersion into refrigerated liquid ethane at approximately 90 K in a temperature-controlled freezing unit (LEICA EM GP, Wetzlar, Germany). The vitrified specimen was transferred to a Zeiss/LEO EM922 transmission electron microscope for imaging using a cryoholder (CT3500, Gatan Inc., Munich, Germany). The temperature of the sample was kept around 90 °K throughout the examination. Specimens were examined under doses of about 100–1000 e/nm^2^ at 200 kV. Images were recorded digitally by a bottom mounted CCD camera (Ultrascan 1000, Gatan, Pleasanton, CA, USA) and subjected to image processing using the system Digital Micrograph GMS 1.9 (Gatan, Munich, Germany).

#### 3.3.2. Photon Correlation Spectroscopy (PCS)

Submicron particle size analysis was performed using a Zetasizer Nano S90 (Malvern Instr., Malvern, England) equipped with a 5 mW helium neon laser with a wavelength output of 633 nm. Plastic-ware was cleaned with detergent washing and rinsed twice with milliQ water. Measurements were made at 25 °C at an angle of 90°. Data were interpreted using the “method of cumulants” [38].

#### 3.3.3. Small Angle X-rays Scattering (SAXS)

Small-angle X-ray scattering (SAXS) data were collected on the bioSAXS beamline B21, at Diamond Light Source (Harwell, UK).

EA loaded and unloaded NLC1 and NLC2 (6 mg/mL) solutions were transferred into 0.2 mL tubes in an automated sample changer. The samples were then delivered into a temperature-controlled quartz capillary and exposed for 1 s, acquiring 30 frames at 20 °C. Data were collected using a Pilatus Dectris 2 M detector with a 3.9 m sample-detector distance and X-ray wavelength λ = 1.0 Å (so that the explored Q-range extended from 0.003 to 0.35 Å^−1^, Q being the modulus of the scattering vector, defined as 4π sin θ/λ, where 2θ is the scattering angle) and corrected for background, detector efficiency, and sample transmission. The two-dimensional (2D) data were then radially averaged to derive I(Q) vs. Q curves.

### 3.4. Drug Content of Dispersions

The total drug content (free plus bonded) of the produced NLC dispersions was determined after subjecting a sample of NLCs to methanol dilution (1:10 *v*/*v*) and a 3 h of stirring, thus destroying the lipid nanoparticles completely. On the other hand, the encapsulation efficiency (EE) of NLCs was determined as follows. One hundred microliters aliquot of each batch was loaded in a centrifugal filter (Microcon centrifugal filter unit YM-10 membrane, NMWCO 10 kDa, Sigma Aldrich, St Louis, MO, USA) and centrifuged (Spectrafuge™ 24D Digital Microcentrifuge, Woodbridge, NJ, USA) at 8000 rpm for 20 min. The amount of entrapped EA was determined by dissolving the lipid phase in the supernatant with a known amount of ethanol (1:10, *v*/*v*), while the amount of free EA was determined in the filtrated aqueous phase. The samples were then filtered through 0.45 μm membrane pore size and analyzed by HPLC as detailed below. All data were the mean of 6 determinations on different batches of the same type of dispersion. EE was determined applying the following equation.

EE = amount of EA detected in the lipid phase × 100/total amount of EA employed(1)

HPLC determinations were performed using an HPLC system Series 1200 (Agilent Technologies Italia, Milan, Italy) equipped with a two-plungers alternative pump (Jasco Corporation, Cremella, Italy) and an UV-detector at 254 nm. Then, 40 μL samples were injected by means of a 7125 Rheodyne injection valve with a 50 μL loop on a stainless steel Kinetex^®^ C18 reverse-phase column (150 mm × 4.6 mm) packed with 5 μm particles (Phenomenex Srl, Milan, Italy). Injections were repeated thrice. Elution of EA was performed with a mobile phase containing methanol (55%), water (45%), and phosphoric acid (0.1%) flowing at a rate of 0.6 mL/min. In these conditions, EA retention time was 5.9 min.

### 3.5. Shelf-Life Studies

Shelf-life stability studies were conducted in triplicate by analyzing at predetermined times, the physical aspect, the drug entrapment, and the size of NLC dispersions up to 2 months from production.

Particularly, physical stability studies were performed analyzing macroscopic aspect (phase separation, turbidity, and macroscopic viscosity) under visual inspection; the drug entrapment was analyzed by HPLC as above described while the size was followed by PCS analyses as above indicated.

Chemical stability was evaluated determining EA content by HPLC analyses (see above), thus shelf-life values were calculated as described by Pugh [39] and summarized below. 

Log (EA residual content, %) was plotted against time allowing the calculation of the slopes (m) by linear regression. Afterwards, the slopes (m) values were used for the determination of k values applying Equation (2).

k = m × 2.303(2)

Shelf life values (the time for 10% loss, *t*_90_) and half-life (the time for 50% loss, *t*_1/2_) were then calculated by means of Equations (3) and (4), respectively.

*t*_90_ = 0.105/k (3)

*t*_1/2_ = 0.693/k (4)

### 3.6. Antioxidant Activity (DPPH and FRAP) 

#### 3.6.1. DPPH (2,2-Diphenyl-1-picrylhydrazyl) Assay

DPPH radical-scavenging assay is widely used to rapidly evaluate antioxidant capacity [40], and in particular it is ideal for phenolic compounds. This assay measures the hydrogen donation ability of an antioxidant to convert the stable DPPH free radical into 1,1-diphenyl-2-picrylhydrazyl, which is accompanied by a colorimetric reaction from deep-violet to light-yellow, which can be evaluated by measuring the percentage reduction of the absorbance of the solution at 517 nm after the radical reaction with the products to be tested. The percentage of radical scavenging capacity was calculated using Equation (5).

DPPH radical − scavenging capacity (%) = [1 − (A1 − A2)/A0] × 100(5)

in which A0 is the absorbance of the control (without EA), A1 is the absorbance in the presence of the EA, and A2 is the absorbance without DPPH. To a methanol solution of DPPH (1.5 mL) 0.750 mL of EA (solution or NLCs in DMSO) at different concentrations were added. The absorbance at 517 nm was measured with a UV–Vis spectrophotometer (Jenway 7305 Spectrophotometer, VWR International Srl, Milan, Italy) according to a described procedure [41]. Results were expressed as µmol Trolox equivalent/g of compounds.

#### 3.6.2. FRAP Assay

The FRAP method is a quantitative assay for measuring the ferric ion reducing ability of plasma and is based on the reduction of ferric ions (Fe^3+^) to ferrous ions (Fe^2+^) under acidic conditions in the presence of 2,4,6-tripyridyl-s-triazine (TPTZ) [42]. In the presence of an antioxidant, the Fe^3+^–TPTZ complex is reduced to the ferrous form, corresponding to an intense blue coloration that is read to a fixed wavelength of the absorption maximum (593 nm). The antioxidant activity is given as µmol Trolox equivalent/g of compounds, as this standard was used to perform the calibration curves.

### 3.7. In Vitro Diffusion Studies

In vitro diffusion studies were performed using Franz-type diffusion cells supplied by Vetrotecnica (Padua, Italy) and associated to 0.45 μm pore size nylon membranes (Merck Millipore, Milan, Italy). Before mounting onto a Franz cell (diameter being 1 cm), nylon membranes were wetted in distilled water at room temperature for 30 min. The exposed membrane area was 0.78 cm^2^. The receiving compartment contained 5 mL of a mixture of ethanol and phosphate buffer 60 mM (30:70, *v*/*v*) alternatively at pH 7.4 or pH 5.5. The solution in the receiving compartment was stirred at 500 rpm with a magnetic bar and maintained at 32 ± 1 °C during the experiments [31,43].

Then, 1 mL of each formulation was placed on the membrane in the donor compartment that was sealed to avoid evaporation. At predetermined time intervals comprised between 1 and 8 h, 0.15 mL of receiving phase were withdrawn and EA content was evaluated by HPLC as above reported. Each removed sample volume was replaced with the same amount of fresh receiving phase. The EA concentrations were determined six times in independent experiments and the mean values ± standard deviations were calculated. The mean values were then plotted as a function of time. The diffusion coefficients, computed from the linear portion of the accumulation curve, represent the experimentally observed fluxes (Jo). Normalized fluxes Jn were then calculated using Equation (6).

Jn = Jo/C(6)

where C is the EA concentration (in mg/mL) of the analyzed formulation.

### 3.8. In Vitro MTT Test

HaCaT cells were grown in Dulbecco’s modified Eagle’s medium (DMEM) high glucose, (Lonza, Milan, Italy), supplemented with 10% FBS (fetal bovine serum), 100 U/mL penicillin, 100 μg/mL streptomycin, and 2 mM l-glutamine. Cells were incubated at 37 °C for 24 h in 95% air/5% CO_2_ until 80% confluence.

The different formulations, namely EA DMSO solution, both types of empty NLCs, NLC-EA1, and NLC-EA2, were dispersed in cell culture medium and diluted to reach EA concentrations ranging from 10 to 75 μM. Concerning empty-NLCs, they were added following the same dilution step used for NLC-EA in order to reach their same content in lipid nanoparticles within the wells.

Seeded cells were exposed to the selected formulations for 24 h, afterwards the treatment was completely removed and 110 μL of MTT (0.5 mg/mL) were added and incubated for 4 h. To convert the MTT solution into a violet colored formazan, 100 μL of DMSO were subsequently added and incubated for 15 min. After shaking, the solution absorbance, proportional to the number of living cells, was measured using a spectrophotometer at 590 nm and, after subtracting background at 670 nm, thus converted into percentage of viability.

### 3.9. Data Analysis and Statistics

Statistical analysis was performed by the analysis of variance (ANOVA). The level of significance was taken at *p*-values < 0.05.

## 4. Conclusions

The low solubility of EA in aqueous solution involves very difficult administration. To ameliorate this, significant amounts of surfactants have to be used resulting in important toxicity in vivo. In this view the possibility to administrate EA using lipid nanoparticles could be very useful and interesting. It was demonstrated that the inclusion of EA within NLCs could improve the water solubility, allowing for a reduction of the dosage. Moreover, the maintenance of high antioxidant effect and low toxicity was evidenced for both types of NLC-EA, even if NLC-EA1 seems better than NLC-EA2. 

It can be concluded that NLCs represent good strategies to encapsulate EA, although further studies aimed at deeply evaluating the absorption/diffusion of EA through the skin have to be carried out. For instance, in vitro studies should be carried out using natural epidermal stratum corneum and/or artificial membranes. In particular, in order to avoid the use of animals, a multi-layered membrane system consisting of a hydrophilic cellulose ester membrane sandwiched between two lipophilic Silastic^®^ membranes should be used. In this way both the lipophilic–hydrophilic structure of human skin and the stratum corneum barrier properties can be satisfactorily reproduced, and in the meantime this system can be useful for simulating the dermal absorption of EA. 

Furthermore, due to the awareness that simulations of human pharmacokinetic parameters and plasma concentration-time curves using in vitro extrapolation in vivo (IVIVE) and physiological based pharmacokinetics (PBPK) models are currently becoming very important and fundamental for the discovery and development of pharmacological processes, these models will be taken into consideration in the future and new experiments will be carried out in order to transpose some of the results obtained in this preliminary study.

## Figures and Tables

**Table 1:** Chemical structure and some physicochemical characteristics of ellagic acid (EA).

Chemical Structure	Molecular Weight	λ_max_ (nm)	Log P	Melting Point (°C)
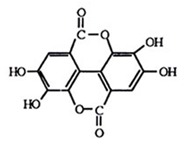	302.20	254	1.59 (2.32)	>360

**Table 2:** Composition of the produced nanostructured lipid carriers (NLC)-EA.

	NLC-EA1 (% of Total Dispersion’s Weight)	NLC-EA2 (% of Total Dispersion’s Weight)
Tristearin	3.35	4
Miglyol	1.65	-
Labrasol	-	1
Aqueous Solution of Poloxamer 188 (2.5% *w*/*v*)	95	95
Ellagic Acid (EA)	0.025	0.025

**Table 3:** Mean diameters of NLCs as determined by photon correlation spectroscopy (PCS).

Day	e-NLC1	e-NLC2	NLC-EA1	NLC-EA2
	Z ave (nm) *P.I.*	Z ave (nm) *P.I.*	Z ave (nm) *P.I.*	Z ave (nm) *P.I.*
1	116.5 ± 0.6 *0.37*	188.3 ± 1.2 *0.31*	195.7 ± 2.1 *0.36*	189.6 ± 3.9 *0.33*
20	118.9 ± 5.6 *0.40*	183.1 ± 6.2 *0.31*	192.0 ± 0.8 *0.52*	181.3 ± 5.1 *0.29*
30	117.2 ± 7.6 *0.43*	182.0 ± 1.5 *0.28*	190.4 ± 2.9 *0.40*	195.8 ± 6.4 *0.37*
60	118.7 ± 2.3 *0.39*	176.6 ± 3.2 *0.33*	189.8 ± 2.3 *0.30*	189.2 ± 2.6 *0.31*

s.d. = standard deviation calculated after five determinations on different batches of the same type of dispersion. P.I.: polydispersity index

**Table 4:** EA content in NLCs as a function of time and shelf-life values.

EA Recovery (%) ^1^			
Time (Days)	EA (Ethanol Solution)	NLC-EA1	NLC-EA2
1	100.00 ± 1.41	91.50 ± 2.42	96.61 ± 3.67
10	96.58 ± 2.22	91.06 ± 3.91	92.69 ± 0.93
20	84.31 ± 2.81	90.37 ± 2.50	91.15 ± 2.27
30	74.29 ± 2.63	89.66 ± 2.51	90.86 ± 1.85
60	48.24 ± 6.14	87.57 ± 2.40	88.63 ± 3.67
**Shelf Life Values**			
***K***	0.012325	0.001832	0.002682
*t*_90_ (days) ^2^	8.52	57.30	39.14
*t*_1/2_ (days) ^3^	56.23	378.18	258.33

^1^: percentage as a function of initial EA content by weight. ^2^: time at which the drug concentration has lost 10%. ^3^: time at which the drug concentration has lost 50%. The results are the average of three independent experiments ± s.d.

**Table 5:** Antioxidant activity of NLC-EA as determined by FRAP and DPPH assays.

Compound *	DPPH		FRAP	
	µmolTE/g ^a^ ± SD	% of Activity ^b^	µmolTE/g ^a^ ± SD	% of Activity ^b^
**EA-solution**	25834.90 ± 0.00	100	34052.21 ± 1902.66	100
**NLC-EA1**	9545.16 ± 0.00	37	19852.21 ± 1419.89	58
**NLC-EA2**	4786.36 ± 112.17	18	20879.52 ± 1981.78	61

* all the compounds were tested at the same concentration (0.005 mg/mL) ^a^: μmol Trolox equivalents/g ^b^: % of activity = percentage of antioxidant activity as compared to EA in DMSO solution.

**Table 6:** In vitro diffusion coefficients of EA.

	Jn (μg/cm^2^/h)	log Jn	R^2^
NLC-EA1 pH 7.4	0.005	−2.30	0.969
NLC-EA2 pH 7.4	0.003	−2.52	0.984
EA-solution pH 7.4	0.112	−0.95	0.990
NLC-EA1 pH 5.5	0.430	−0.36	0.979
NLC-EA2 pH 5.5	0.500	−0.30	0.995
EA-solution pH 5.5	0.663	−0.17	0.999
